# Current and Emerging Therapies to Combat Cystic Fibrosis Lung Infections

**DOI:** 10.3390/microorganisms9091874

**Published:** 2021-09-03

**Authors:** Jim Manos

**Affiliations:** Infection, Immunity and Inflammation, Faculty of Medicine and Health, School of Medical Sciences, The University of Sydney, Sydney 2006, Australia; jim.manos@sydney.edu.au

**Keywords:** cystic fibrosis, lung infection, CF bacteria, antibiotic, combined therapy, novel therapy, CFTR modulator

## Abstract

The ultimate aim of any antimicrobial treatment is a better infection outcome for the patient. Here, we review the current state of treatment for bacterial infections in cystic fibrosis (CF) lung while also investigating potential new treatments being developed to see how they may change the dynamics of antimicrobial therapy. Treatment with antibiotics coupled with regular physical therapy has been shown to reduce exacerbations and may eradicate some strains. Therapies such as hypertonic saline and inhaled Pulmozyme^TM^ (DNase-I) improve mucus clearance, while modifier drugs, singly and more successfully in combination, re-open certain mutant forms of the cystic fibrosis transmembrane conductance regulator (CFTR) to enable ion passage. No current method, however, completely eradicates infection, mainly due to bacterial survival within biofilm aggregates. Lung transplants increase lifespan, but reinfection is a continuing problem. CFTR modifiers normalise ion transport for the affected mutations, but there is conflicting evidence on bacterial clearance. Emerging treatments combine antibiotics with novel compounds including quorum-sensing inhibitors, antioxidants, and enzymes, or with bacteriophages, aiming to disrupt the biofilm matrix and improve antibiotic access. Other treatments involve bacteriophages that target, infect and kill bacteria. These novel therapeutic approaches are showing good promise in vitro, and a few have made the leap to in vivo testing.

## 1. Introduction—The Cystic Fibrosis Lung Environment

Cystic fibrosis (CF) is the most common life-threatening autosomal recessive genetic disease in the Caucasian population, with lower yet still significant incidence in other racial populations. There are an estimated 100,000 people living with CF worldwide, and the disease has an incidence of one in 2500–4000 amongst Caucasians [1]. The genetic defect that gives rise to CF occurs in the gene coding for the CF transmembrane conductance regulator (CFTR) located on chromosome 7, where specific amino acid mutations can change the conformation of the protein resulting in a closed or non-functional CFTR. The CFTR is a membrane protein forming a channel that conducts chloride ions across the epithelium, and this movement does not occur or is greatly reduced in CF depending on the mutation [2]. This loss of chloride results in the CF lung mucus becoming more viscous, less likely to detach from submucosal glands and thus more difficult to expel via mucociliary clearance [3]. Furthermore, the impairment of the CFTR also affects bicarbonate (HCO_3_^−^) secretion [4]. The lack of this alkaline balance leads to a lower pH in the CF lung compared to normal lung.

Airway surface liquid, which coats the epithelium in two layers: an aqueous layer adjacent to the surface, and a gel or mucous layer above it, is also impacted by the impaired CFTR. In the non-CF lung, airway surface liquid acts as a first line of defence against infection [5], with the gel layer mucins trapping bacteria and parasites for later expulsion and killing them through the actions of innate immune system antimicrobial peptides. In the CF lung, the airway surface liquid is acidified by loss of HCO_3_^−^, and the gel layer is consequently more viscous, similar to that of mucus [6]. This lack of mobility impairs the action of antimicrobial peptides such as the cationic host defence peptide LL-37, which has been demonstrated to be a potent biofilm inhibitor, inhibiting *Pseudomonas aeruginosa* and *Staphylococcus aureus* biofilm formation in vitro [7,8]. Therefore, loss of this first line of defence would have consequences for the establishment of infection in the CF lung.

Hypoxic conditions in deep mucus also drive bacterial growth. CF mucus can plug the smaller airways semi-permanently. Oxygen tension within mucus plugs has been shown to fall from ca.160 to almost zero mm Hg [9], and this change would be expected to favour facultative or anaerobic bacteria, while hindering immune cell responses. Induction of hypoxia-inducible factor (HIF-1) does not appear to reverse this loss of immune function [10]. CF mucus is also very nutrient rich, containing greater concentrations of amino acids and iron than non-CF mucus. In cases of severe CF lung disease dominated by *P. aeruginosa* and its protease activity, the levels of phenylalanine and tryptophan have been shown to be significantly higher than in the mucus of less severe lung disease not dominated by *P. aeruginosa* [11]. In contrast, the mucus of CF patients not dominated by *P. aeruginosa* contained numerous disparate species, including anaerobes such as *Streptoccoccus* sp., *Prevotella melaninogenica*, and *Veillonella dispar.*

## 2. Infecting Species by Age Cohort

**Figure 1:**
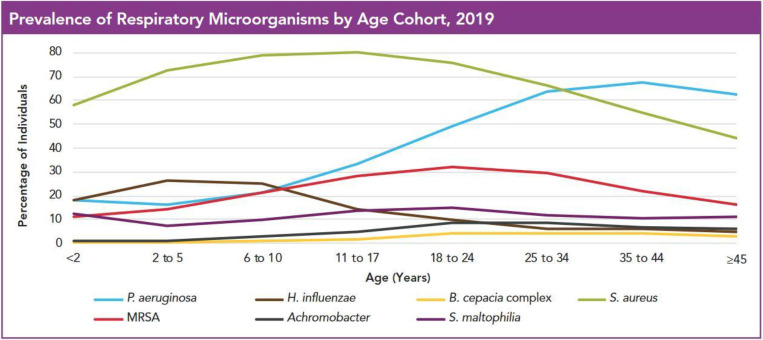
Prevalence of respiratory bacteria in the lungs of cystic fibrosis patients—by age cohort. From the US CF Foundation Annual Data Report 2020. Data source: Cystic fibrosis patients under care at CF Foundation-accredited care centres in the United States, who consented to have their data entered [12].

When bacterial prevalence is measured by age cohort, an obvious change in infective species isolated becomes apparent through the patients’ lifetime. The US Cystic Fibrosis Foundation data registry statistics for 2019 (Figure 1) demonstrated that by far the most prevalent species isolated from sputum in the first five years of life of CF patients was *S. aureus* (70% methicillin-sensitive (MSSA)), while *Haemophilus influenzae* and *P. aeruginosa* were in second (~28%) and third (~18%) place, respectively. In the 6 to 10-year age group, there is little change. *S. aureus* continues to dominate, with percentages of both methicillin-resistant (MRSA) and MSSA rising to ca. 80% and 20%, respectively, while *H. influenzae* begins to fall and *P. aeruginosa* increases slightly to ~25%.

### 2.1. The Challenges of S. aureus Infection in Pre-Adolescents

The main clinical challenge in infants and children is *S. aureus*. Colonisation with *S. aureus* (mostly methicillin sensitive *S. aureus* (MSSA)) occurs in the first days of life, as more than 70% of newborns with CF have basal *S. aureus*-positive cultures. The first weeks of life are also critical for the emergence of persistent infection. Newborn screening of infants, introduced in the early 2000s, has resulted in a greater understanding of the progress of early infection and its effects on lung health. In infants, studies have shown that pulmonary inflammation correlates with lower lung function, whereas pulmonary infection correlates with a greater rate of decline in lung function [13]. Therefore, the presence of a persistent infection in infancy already appears to predetermine worse clinical outcomes over time.

While culture-based studies have historically shown high carriage rates in infants, the data obtained is dependent on the sample and the sampling site. A study by Esposito et al. [14] showed that of 264 *S. aureus* carriers aged 6 to 17 years, 39.2% were nasal carriers, 25.9% oropharyngeal carriers and 12.1% had infection in both sites. These results were confirmed by use of PCR for identification of *S. aureus* from infant nasal samples [15] which demonstrated an increase from 16.2% infected at two months to 21.1% infected at four months.

Although MSSA has continued to make up the bulk of strains isolated from children, the prevalence of MRSA has been increasing in the US and South America, while remaining steady across most of Europe and in Australia. Potential risk factors in acquisition of hospital-acquired strains of MRSA (HA-MRSA) include exposure to other CF patients in hospital settings and clinics and surgery. For community acquired MRSA, risk factors include acquisition from siblings or family members [16,17]. 

### 2.2. Non-Typable Haemophilus Influenzae and H. parainfluenzae—A Significant Childhood Threat

*H. influenzae* and *H. parainfluenzae* are major colonisers of CF lung, particularly in children and adolescents. *H. influenzae* is considered a commensal inhabitant of the healthy nasopharynx, however the factors that lead to increased risk in CF patients remain largely undefined. What is clear is that *H. influenzae* opportunistically attaches to the CF airway epithelia, aided by slow mucus clearance, and readily forms biofilms [18].

A vaccine targeting the encapsulated strain of *H. influenzae* b (the most pathogenic encapsulated variety) has proven very effective. However, there is no vaccine against non-encapsulated strains, termed non-typeable *H. influenzae* (NTHi). NTHi has invasive properties that are particularly harmful in lung infection [19,20], with evidence that NTHi may invade the bronchial wall to activate T cells and contribute to inflammation. NTHi has been shown to survive inside mononuclear phagocytes and epithelial cells for 72 h and to invade lung parenchyma [21]. 

The few published studies show a prevalence of 20–25% in children and adolescents. A large patient cohort study by Cardines et al. (n 300 median age = 15.4 years) showed a prevalence rate of 21.3%, and this is in accordance with previously published studies [22]. A retrospective longitudinal study of 349 patients over 15 years (1998–2012) indicated a doubling of prevalence (8–16%) for *H. influenzae* (*p* < 0.001; mean age 7.6 years) and a 10-fold increase in prevalence for *H. parainfluenzae* (*p* < 0.0001; mean age 8.6 years) over the entire period. The annual increase in prevalence for *H. parainfluenzae* was greater from 2008 onwards (13 to 40%), indicating that this species is likely becoming a greater cause of infection in younger CF patients [23]. 

### 2.3. P. aeruginosa—Slow to Start but Bound to Dominate

A number of studies over the past two decades have shown that infection with *P. aeruginosa* in the first decade of life to results in a more rapid decline in lung function, increased exacerbations and hospitalisations and a more rapid progression to early death. The progression to chronic *P. aeruginosa* infection presents as a marker of this decline [13,24,25,26,27]. *P. aeruginosa* is also a prolific biofilm former, with cells persisting within biofilms despite repeated rounds of antibiotic treatment. With the introduction of ‘easy to use’ inhaled antibiotics in the early 2000s, the paradigm has shifted from support with physical therapy during exacerbations, to early intervention and treatment to eradicate the infection. While guidelines for inhaled antibiotics have yet to be established for children under six years of age, the recommendation to “treat early and treat for eradication” has been adopted in the US and Europe [28]. In addition to its potential for chronic infection, the mucoid (alginate-overexpressing) phenotype is a known predictor of poorer outcomes [29,30,31]. A recent large cohort study of longitudinally acquired data showed the prevalence of mucoid *P. aeruginosa* rises steadily with age, from 1% at age one to 8% at age nine [32]. Furthermore, of the 3580 children with *P. aeruginosa*, 17% had a mucoid phenotype while 13% went on to develop chronic infection, and Cox regression analysis showed a significant association between mucoidy and the development of chronic infection (Hazard Ratio = 2.59, 95% CI 2.11, 3.19) [32]. 

Therefore, to avoid emergence of mucoid strains and chronic infection, the emphasis on clearing *P. aeruginosa* infection early is now established in European countries, Australia and North America [27,33,34]. These measures are having an impact on childhood prevalence of *P. aeruginosa* and other infecting species. The US CFF registry data for 2019 recorded a decline from 44.8% to 25.1% in the period 1999–2019 for *P. aeruginosa* isolation from CF patients under 18 years of age [12]. An 18-year follow-up of 380 children first diagnosed via newborn screening at two Australian CF centres showed a significant decrease in prevalence of *P. aeruginosa* from 12.3% in the period 2000–2006 to 5.9% in the period 2012–2018 (*p* < 0.001). *S. aureus* prevalence also fell from 15.7% to 9.1% in the same period (*p* < 0.001). This decrease was evident in all age groups (0–2, 3–4 and 5–6 years) when comparisons were made between the time periods 2000–2006, 2006–2012 and 2012–2018; and was consistent across both CF centres [35]. By linking results to the introduction of new antibiotic regimens and therapies, the authors were able conclude that the decrease in prevalence was directly related to the change toward more aggressive treatment.

Failure of aggressive therapy to eradicate *P. aeruginosa* is an ongoing problem and has occurred in as much as 49% of a patient cohort, according to a study of 21 eradication therapies [36]. Delay in the detection of *P. aeruginosa* and lack of timely initiation of treatment may limit the success of the intervention [36]. Mucoid status of the *P. aeruginosa* isolate has been previously associated with eradication failure.

Pinning down virulence factors associated with childhood strains of *P. aeruginosa* has proved somewhat more elusive. Pathogenic progression of acute infection depends on a functional type 3 secretion system (T3SS) [37] and significantly raised serum antibody concentrations to specific components have been identified [38]. However, longitudinal studies show a decline in virulence factor expression over time. A study of 12 virulence factors (pyocyanin, pyoverdine, elastase, phospholipase C, total protease, swarming, rhamnolipid, haemolysin, biofilm mass, swimming, twitching and colony size) in samples taken from 168 CF infants and children between newborn screening and 36+ months (Table 1) showed that the longer the time since initial isolation of a strain, the lower the level of virulence factor expression [39]. 

Some evidence for virulence differences has emerged from studies of persistent strains where intensive inhaled therapy has failed [40]. A comparison of 65 eradicated and 21 persistent *P. aeruginosa* morphotypes showed the latter had significantly reduced twitching motility and mucoidy (*p* < 0.001 and 0.002, respectively), as well as a significantly higher tobramycin MIC (≥128 μg/mL) [40]. 

At the genetic level, the long-held notion of convergent evolution through host adaptation has been reinforced by sequence comparisons that show a remodelling of the genetic pathways of virulence genes during infection. A study of the genomes of 474 isolates of *P. aeruginosa* from 34 children and young adults (median age at first sequenced *P. aeruginosa* isolate = 8.8 years, range = 1.4–26.3 years) demonstrated convergence of 52 genes, many of which are known virulence factors involved in mucoidy, multidrug resistance, LPS/capsule, alginate, pilin and phenazine biosynthesis [41]. 

### 2.4. Acquisition Prevention and Early Eradication Strategies

Strategies used for prevention of acquisition include cohort segregation, individual segregation, hand hygiene, facemasks, combination strategies, equipment strategies, and adherence to medication regimens. Cohort and individual segregation, when the uninfected are segregated from those with *P. aeruginosa* by ward as inpatients and by different visit day as outpatients, show evidence for lower transmission and acquisition [42,43]. Significant reductions were especially evident with clonal strains [44,45]. However, strong arguments exist that segregation must be coupled with infection control measures such as hand hygiene and facemasks to be most effective [46]. 

Studies have demonstrated that early eradication is effective in delaying chronic and mucoid *P. aeruginosa* infection. A 5-year observational study of 296 children being treated for their first *P. aeruginosa* infection by Mayer-Hamblett et al. showed that those negative by culture for ≥12 months after commencement of antibiotic therapy had a 74% reduced risk of developing chronic *P. aeruginosa* (hazard ratio [HR], 0.26; 95% CI, 0.17–0.40) and a 57% reduced risk of mucoidy (HR, 0.43; 95% CI, 0.25–0.73) compared with those that were not negative at 12 months [47]. While promising, studies such as these likely require a longer observation time to clearly identify the transition from acute to chronic and mucoid infection.

### 2.5. Surveillance

Periodic surveillance strategies have been developed to identify and map infections within a community. Untargeted surveillance predates genotyping and includes lipopolysaccharide serotyping to identify differences in the *P. aeruginosa* O-polysaccharide, phage typing and pulsed-field gel electrophoresis (PFGE), the last considered the “gold standard” in genotyping of clinical isolates [48]. Whole-genome sequencing allows targeted surveillance through quantitative-PCR assays for strain-specific genes, and these have been successfully adapted to assay sputum directly, thus enabling the earlier detection of infecting strains compared to culture [49]. This has enabled more timely antibiotic interventions with a stronger possibility of eradication, though a cautious approach to a positive qPCR is required, as presence of amplified DNA or RNA does not necessarily indicate an active infection. 

## 3. Species and Prevalence in Adolescence and Young Adulthood

### 3.1. Changes in Pre-Existing Species

With increased antibiotic therapy, the general diversity of bacterial species in the CF lung begins to decline in adolescence and young adulthood (ca. 12–24 years), with a core set of recalcitrant species remaining in older adults [50]. The most obvious change that occurs is the decline in prevalence of *H. influenzae,* with the US statistics (Figure 1) showing a decline from ca. 25% to ca. 10%. Australian CF data by respiratory culture show a similar decline, from 33.6% and 23.5% in the 2–5 and 6–11 year age groups, respectively, to 8.1% in the 18–29 year age group [51]. The decline can be attributed to eradication therapy and to the vaccines available since 1992 against *H. influenzae* type b, which have largely eliminated the prevalence of this infectious cohort. Three conjugate polysaccharide vaccines are licenced; they differ in the size of the polysaccharide, the chemical linkage between the polysaccharide and the carrier, and the type of protein carrier used [52]. The NTHi cohort makes up almost all remaining cases in children and adults, and the inherent heterogeneity of this group has so far prevented development of a useful vaccine.

The US Cystic Fibrosis Foundation data show that while the overall *S. aureus* prevalence peaked in adolescence and showed a modest decline in the 18–24-year age group, the prevalence of MRSA remained steady at ca. 30% between the ages of 11 and 34, highlighting the seriousness of this persistent infector in the US. The prevalence of MRSA in Australia is much lower, though also steady, for this age group (4.5 and 5.0% in the 2–17 and 18–29-year age groups, respectively) [51]. Europe-wide data on MRSA prevalence in adolescents are hard to come by. However, studies show that prevalence rates are high, though lower than that shown in the US data. In the annual reports of the European CF foundation, the rate amongst those over 18 was 16% in 2007 and remains above 25% across all groups in seven of the 29 reporting countries as of 2018 [53].

The increasing prevalence of *P. aeruginosa* in adolescence and young adulthood is the most obvious consequence of the eradication of other species in earlier years of life, leading to a drop in lung species diversity [54,55,56]. The US CFF data (Figure 1) clearly show that the rise in *P. aeruginosa* prevalence from ca. 20% in the 6–10-year age group to ca. 50% in the 18–24-year age group is paralleled by a drop in *H. influenzae* prevalence from 25% to ca 10%, and the beginnings of a fall in *S. aureus* (MRSA and MSSA) prevalence. The Australian CF data show an even steeper rise in *P. aeruginosa* prevalence, from 31% in the 6–11 -year age group to 69.9% in the 18–29 -year age group, with the concomitant falls in *H. influenzae* prevalence described above [51]. While the data show a link in prevalence levels, there is no evidence to date of a link with increased disease severity or pathogenicity of *P. aeruginosa* infection.

### 3.2. Achromobacter xylosoxidans and Stenotrophomonas maltophilia

The adolescent CF lung begins to show a greater, though still subdued, prevalence for four other species compared to the CF child lung: *Stenotrophomonas maltophilia*, *Achromobacter xylosoxidans* (formerly *Alcaligenes xylosoxidans*) *Burkholderia cepacia* complex (BCC) and non-tuberculous Mycobacterium ((NTM) encompassing all Mycobacterial species other than *Mycobacterium tuberculosis* and *Mycobacterium leprae*. *A. xylosoxidans* is an emerging CF pathogen with acquired resistance to a range of antibiotics and an ability to form robust biofilms [57,58], thus limiting eradication strategies. While there is evidence that other species of Achromobacter such as *Achromobacter ruhlandii* are also present in CF lung, their incidence is low and tends to show geographic diversity [59]. *A. xylosoxidans* comprise the overwhelming majority of isolates, and US CFFUS CFF and other registries do not as yet report Achromobacter to the species level. *S. maltophilia* becomes increasingly common in adolescents. By adulthood, it is the third most common multidrug-resistant species identified as the cause of pulmonary infections after *P. aeruginosa,* and MRSA [60]. Infection with *S. maltophilia* is associated with increased pulmonary exacerbations, higher mortality, and may require lung transplantation [61].

The US CFFUS CFF data (Figure 1) show the prevalence of *A. xylosoxidans* rising from ca. 2% at 6–10 years to ca. 10% by 18–24 years, while *S. maltophilia* increased from ca. 10% to ca. 18% in the same age groups. The Australian CF data also showed a rise for *A. xylosoxidans* from 2.3% at 6–11 years to 7.8 % at 18–29 years. Interestingly, the same data showed *S. maltophilia* decreased from 12.2% to 8.8% over the same period after peaking at 14% in the 12–17-year age group [51].

### 3.3. Burkholderia Cepacia Complex (BCC) and Non-Tuberculous Mycobacterium (NTM)

While BCC is generally found at quite low levels throughout the life of most CF patients, there is a slight increase in prevalence in adolescents peaking at approximately 5% in the 18–24-year age group in the US CFFUS CFF data (Figure 1). The Australian CF data showed that BCC doubled from 1.9% to 4.0% in the same age groups. Although prevalence is low compared to other species, infection with BCC poses risks of respiratory failure and rapidly declining lung function as infection with *B. cenocepacia*, particularly the ET12 strain, can cause fulminating, often fatal, pneumonia and septicaemia (“cepacia syndrome”) [62]. While rates have fallen due to stricter infection control procedures, necrotising pneumonia contraindicates lung transplants due to high failure rates [63]. 

NTM has emerged as a major threat to the health of individuals with CF but remains difficult to diagnose and problematic to treat. Verification through testing has proved inconsistent and at times unreliable for those under 10 years of age but US CFF data for specific NTM species in the period 2010–2012 show a prevalence of 11.2% and 12.8% for 11–17 and 18–25-year-olds, respectively [64]. Other studies show a general increase in prevalence with age; with 10% of 10-year-olds infected compared to 30% of those aged over 40 [65]. Australian CF data showed that NTM species increased from 1.8% in the 2–5-year age group to 9.0% in the 12–17-year age group, after which they fell to around 4% in the 18–24-year age group [51].

## 4. Species and Prevalence in Adulthood 

The most dramatic change in species prevalence in the adult CF population, compared to infants, children and adolescents, is the increase in *P. aeruginosa* prevalence. In the US CFF data (Figure 1), prevalence doubled from ca. 35% in the 11–17-year age group to ca. 70% in the 35–44-year age group. This increase has been consistent in data reports from all reporting countries since the 1990s.

The other significant change in adulthood is the decline in *S. aureus* (both MSSA and MRSA) prevalence. After peaking in the 11–17-year age group at ca. 80% for MSSA and 30% for MRSA, the prevalence fell gradually to 45% and 20%, respectively, in the ≥45-year age group.

Several factors combine to give *P. aeruginosa* a competitive advantage. The increase in mucus secretion and impaired mucociliary clearance [66] becomes more pronounced in adulthood and this abundance of mucus assists pulmonary microbial communities in CF to flourish. The advantage to *P. aeruginosa* in this environment can be largely attributed to two factors: antibiotic resistance and genetic diversity.

Antimicrobial therapy is a lifelong experience for CF patients. The effects of prolonged antibiotic therapy on *P. aeruginosa* populations in the CF lung are becoming clearer through recent studies. Phenotypic changes in *P. aeruginosa* populations in 14 CF patients before and after antibiotic treatment were investigated by Fernandez-Barat et al. [67]. There was no significant difference in biofilm size before and after therapy (355.12 [75.79–893.56] vs. 427.68 [244.27–630.41], *p* = 0.75, respectively), indicating biofilm formation persists despite intensive antimicrobial therapy.

Hypermutator phenotypes represent ca. 22–30% of *P. aeruginosa* isolates in patient studies from Europe, North America and Australia [68,69,70,71] and result from the mutation of genes involved in DNA repair. Particularly mutable genes include those involved in mismatch repair such as *mutL*, *mutS* and *uvrD*. Adaptation of *P. aeruginosa* to the CF lung leads to a number of phenotypic and gene mutation changes that may be accelerated in the hypermutator phenotype. Phenotypic changes include conversion to the mucoid phenotype and loss of motility, while mutational changes include upregulation of β-lactamase [72] and efflux pump expression [73], with cumulative effects leading to multidrug resistant strains. 

In vitro studies in artificial sputum medium support the rapid evolution of hypermutator phenotypes [74] and the speed of this evolution may be connected to the level of pressure of antibiotic treatment. Klockgether et al. [75] recently demonstrated that not all mutation levels are equal. A longitudinal study of the initial persisting *P. aeruginosa* clone from patients with mild CF for over 15–25 years, and from CF patients where a fatal outcome occurred within 15 years, showed that the most widespread and debilitating mutations (e.g., those leading to loss of function) occurred in strains from patients with a fatal outcome, whereas changes resulting in greater metabolic versatility were most prominent in strains from CF patients with mild CF. 

The high overall *S. aureus* prevalence appears to be a US phenomenon and possibly related to the antibiotic treatment regimen of CF patients in that country. The European Cystic Fibrosis Society Patient Registry data for the period 2011–2016 shows no change between the 11–17 and 18–25-year age groups (ca. 44%) while a fall to ca. 30% is evident by the ≥40-year age group [76]. In the period 2011–2016, the prevalence in the oldest cohort (≥40 years) remained relatively stable (28.52% to 32.13%). In contrast, the US Cystic Fibrosis Foundation data for the same period showed MSSA and MRSA at ca. 43% and 20%, respectively, in 2011 and ca. 48% and 20%, respectively, in 2016 [12].

### Differences in Treatment Strategies between Age Groups

The most obvious clinical change that impacts infection treatment in CF patients is the age-related progression to chronic infection. Acute and persistent lung infections in infants and young children are regularly eradicated by carefully prescribed antibiotic regimens. However, as the lungs develop, mucus levels increase and the effectiveness of antibiotic clearance decreases, leading to chronic infection. A second development, clearly demonstrated by Figure 1, is the change in species composition, with *P. aeruginosa* becoming the dominant species and *S. aureus* in second place by adulthood [77]. The isolates of *P. aeruginosa* and *S. aureus* recovered from adults are also more likely to be multidrug resistant and originating from biofilm aggregates within the mucus. Additionally, the existence of subpopulations of persister cells, cells with increased tolerance to antibiotics, complicates antibiotic treatment [78]. While persister fractions are low in environmental populations, the highest subpopulations of persister cells have been identified in chronic infections of all types [79], leading to the conclusion that increased persister cell levels are a side effect of long-term antibiotic treatment. Furthermore, the convergence of persister cells and biofilm growth in chronic infection is mutually beneficial to bacterial persistence.

In addition to antibiotic regimens, a form of physical therapy is recommended (see below) to mobilise mucus for expulsion. *P. aeruginosa* various regimens are available, including 28 days of inhaled tobramycin and up to three months of a combination of nebulised colistin and oral ciprofloxacin. Failure of this eradication therapy leads to chronic infection with neutrophil-driven inflammation and episodes of acute exacerbation, following which lung function may fail to return to baseline levels.

In adolescents and adults, treatment is long term (months to years, rather than days to weeks) and often combined with DNase-I (dornase alfa) or hypertonic saline to enhance mucus penetration. The sputum is rendered less viscous in a dose-dependent fashion, leading to better expulsion through coughing [80]. Antibiotics include dry powder inhaled tobramycin or aztreonam with the latter recommended as an alternative by both European and US guidelines. Inhaled colistin is also widely used [81]. Alternating antibiotics monthly reduces development of further resistance (see continuous alternating inhaled therapy). Inhaled antibiotic therapy has been shown to decrease exacerbations and improve lung function [82]. Overall, while lung function can be improved and exacerbations reduced, bacterial eradication is unlikely to be achieved. Reasons for failure include permanent lung damage preventing proper inhalation and poor antibiotic penetration of biofilm aggregates.

## 5. Current Treatments for Bacterial Infection in CF

### 5.1. Non-Antibiotic Treatments—Physical Treatments to Promote Airway Clearance

Physical methods to expel mucus also remove bacterial aggregates and have been used in conjunction with antibiotic treatment worldwide. Chest physical therapy has been the gold standard in airway clearance for adult CF patients, acting as an adjunct to antibiotic therapy. However, benefits are dependent on the correct application of the method. A recent review of physical therapy versus no therapy [83] found that only one of four studies demonstrated significant improvement in pulmonary function following physical therapy intervention, compared to controls. Procedures have been refined to reduce the need for labour-intensive sessions and include the active cycle of breathing technique (ACBT), the forced expiration technique (FET) and exercise routines. Airway clearance techniques in general provide short-term increases mucus transport, with no evidence of long-term effects [83]. These should not be performed in children as a routine intervention or for acute infection [84]. Improved inhalation methods for antibiotic treatment have led to a reduction in the use of physical therapy for some patients, but there is strong evidence for its effectiveness when used correctly in parallel with antibiotic treatment. The main treatments are:

#### 5.1.1. Postural Drainage

Studies show postural drainage to be most effective in treating impaired mucociliary clearance [85,86], and that three days of postural drainage treatment results in statistically significant improvements in pulmonary function [87].

#### 5.1.2. Percussion Therapy

There are six recommended percussive chest physiotherapy positions and it is critical that the positioning be maintained for the entire percussion period (three to five minutes) [88] Its overall effectiveness as a treatment is similar to postural drainage. However, it is long (20–40 min), labour intensive and tiring for the patient.

#### 5.1.3. Forced Expiratory Methods (FET and PEP)

FET consists of one or two forced expirations, or huffs, followed by relaxation and breathing control. Its effectiveness appears to be greater in adults than in children [89,90]. The main problem with FET and associated expiratory techniques is lack of regular use by clinic attendees, or low patient compliance if undertaken at home. The positive expiratory pressure (PEP) device provides back pressure to the airways during expiration and improved clearance by accumulating gas behind mucus and separating it from the airway wall. A review of 28 studies involving 788 participants found that the efficacy of PEP was similar to other methods of chest physiotherapy including postural drainage with percussion, active cycle of breathing techniques, and autogenic drainage, with no decisive advantage to PEP over other methods [91].

#### 5.1.4. ACBT 

ACBT uses a cycle of steps to loosen airway secretions including breathing control and thoracic expansion exercises and is usually coupled with the FET. A recent Cochrane review found no significant difference in quality of life, sputum weight, exercise tolerance, lung function, or oxygen saturation between the active cycle of breathing technique and other methods such as FET and percussion therapy [92].

#### 5.1.5. Exercise Training

Exercise training aims to alter respiratory muscle strength and endurance, improve the perception of dyspnoea, and increase secretion clearance in CF patients, with improvements in pulmonary function and a possible training effect on respiratory muscles [93]. There is strong interest by physicians but a lack of suitable testing programs. Consequently, it is often put in the “too hard” basket in favour of other treatments [94].

### 5.2. Antibiotics

Antibiotics form the mainstay of bacterial infection treatment from infancy. Most antibiotic treatments after adolescence are focused on the main infecting species and the greatest contributor to morbidity and mortality, *P. aeruginosa*, and most research has been directed towards treatments for this infection. Antibiotics may be administered orally, as inhaled or nebulised particles or injected intravenously. Treatment times traditionally depended on the level of severity of the infection and whether a pulmonary exacerbation (defined as episodic increases in respiratory symptoms such as cough and sputum production and systemic symptoms such as fatigue and weight loss) had occurred. In adults, oral antibiotics are usually prescribed to treat less severe pulmonary exacerbations, and this may in fact be detrimental to the patient’s long-term % predicted FEV_1_. Wegener et al. [95] showed that a short-term improvement in % predicted FEV_1_ was significantly greater for pulmonary exacerbations treated with IV antibiotics compared to non-IV treatments (5.1% ± 12.7 vs. 2.0 ± 11.6 %-predicted, *p* < 0.001). These data from the period 2003–2005 also showed that almost half of the pulmonary exacerbations (45.5%) were treated with oral antibiotics alone, while 20% were treated with IV alone and just 1.9% were treated with inhaled antibiotics alone. 

**Figure 2:**
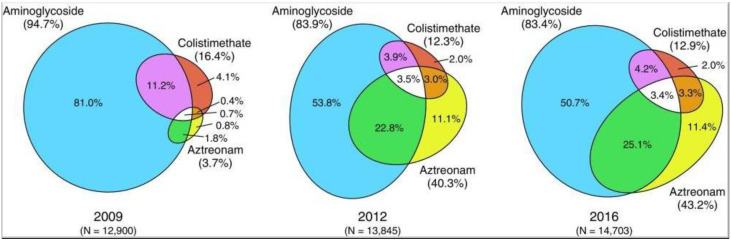
Shifting patterns of inhaled antibiotic class use in the USA. Area-proportional diagrams of proportions of patients receiving inhaled antibiotics in 2009, 2012 and 2016. Reprinted with permission of the American Thoracic Society. Copyright© 2021 American Thoracic Society. Nicholls, D.P et al., 2019, Developing inhaled antibiotics in cystic fibrosis: Current challenges and opportunities. *Annals of the American Thoracic Society* 16 (5): 534–539 [97].

Over the last two decades, inhaled antibiotics have become the preferred option, and studies have demonstrated improved pulmonary function and reduced exacerbation rates for inhaled over oral antibiotics [96]. Three inhaled antibiotics are currently in use for treating *P. aeruginosa*: tobramycin (an aminoglycoside), colistin (a polymyxin) and aztreonam (a monobactam). Preferences for the type of antibiotic have changed since the release of aztreonam. US Cystic Fibrosis National Patient Registry data (Figure 2) show a shift away from aminoglycoside alone (81% in 2009 to 50.7% in 2016) and increasing use of monobactams such as aztreonam (3.7% to 43.2% in the same period).

All three antibiotics have been independently linked to increased lung function, increased body weight and reduced need for hospitalisation [98,99,100,101]. Additionally, the treatment regimen has changed to one of rotation of antibiotic therapies to reduce the emergence of resistance. 

Continuous alternating inhaled antibiotic therapy, where two antibiotics are inhaled in alternating cycles for an extended period of time (weeks to months), has become the norm, irrespective of patient status. Studies have shown that continuous alternating inhaled antibiotic therapy can result in a noticeable improvement in % predicted FEV_1_ compared to intermittent use of antibiotics at time of exacerbation. A single-centre study by Van de Kerkhove (2016) tracked the change in % predicted FEV_1_ for 89 patients (49 on continuous alternating inhaled antibiotic therapy and 40 on intermittent use of antibiotics) over an 18-month period [102]. Use of continuous alternating inhaled antibiotic therapy in the 49 patients was associated with a significant yearly improvement in FEV_1_ compared to the patients on intermittent use of antibiotics (1.148 per year (1.038/0.904 for intermittent use of antibiotics) (95% CI: 1.068–1.236, *p* = 0.0002 versus before continuous alternating inhaled antibiotic therapy in paired analysis)).

Dry powder inhalation has become an alternative to inhaled antibiotic treatment in the last decade. A study comparing tobramycin administered as an inhaled powder against administration as an inhaled solution indicated dry powder inhalation may have an advantage over inhaled solutions in terms of patient satisfaction by shortening the administration time and eliminating the burden of nebuliser maintenance, and thus increasing patient adherence [103]. However, the inhalation procedure is not completely passive and correct inhalation is essential for effective use. Deep exhalation followed by deep inspiration, with a time-dependent holding of the breath, is essential to effective uptake of the powder [104]. While adults can be in a better position to adhere to these requirements, they make dry powder inhalation particularly challenging as a treatment for children. 

### 5.3. Hypertonic Saline

Hypertonic saline as a nebulised mucociliary clearance treatment using 7% *w*/*v* sodium chloride has been available since the mid-1990s. Hypertonic saline works on the principle that inhaled saline rehydrates the dehydrated mucus of the CF airways and improves secretion flow. It may be used alone or in an admixture with an antibiotic. It has been effective at reducing the frequency of pulmonary exacerbations, and studies of its efficacy have usually also indicated an improvement in baseline % predicted FEV_1_ after four weeks of treatment. A year-long, double-blind, parallel-group trial of 164 patients with stable cystic fibrosis demonstrated improved lung function, a marked reduction in exacerbation frequency and a reduced requirement for antibiotic intervention [105]. Evidence also suggests hypertonic saline could be an effective adjunct to physical therapy during acute exacerbations of lung disease in adults. However, there are gaps in its usefulness, as a recent Cochrane review of several hypertonic saline studies showed that the % predicted FEV_1_ improvement is usually not sustained, and hypertonic saline is not effective in children under the age of six [106], though a 2019 study showed significant improvement from baseline in infants [107]. Admixtures of colistin and 5.85% NaCl (as hypertonic saline) are compatible when mixed immediately before administration [108], but no information exists on whether site delivery was affected.

### 5.4. Combined Treatments with Antibiotics 

While antibiotics have been combined or used alternately in treatment regimens for CF infections since the 1980s, and continuous alternating inhaled therapy (discussed above), has proved significantly efficacious, the combination of other compounds such as enzymes, bacteriophages and antioxidants with and without antibiotics, has also become a major area of research and development in treating CF infections. One reason for this focus is that infecting bacteria tend to form microcolonies and aggregates akin to small biofilms in CF lung mucus and lung epithelial cells and antibiotics are unable to comprehensively penetrate these biofilms, leading to passive resistance. This has led to a search for compounds that act on the biofilm matrix components and disrupt them as a prelude to better antibiotic action. The most widespread and arguably most effective combination of antibiotic and another compound currently in use is the coupling of the enzyme deoxyribonuclease-I (DNase-I or dornase alfa) manufactured and marketed as Pulmozyme^®^ by Genentech Inc. with antibiotic such as tobramycin. Dornase alfa used alone is considered a mucociliary clearance treatment through enzymic digestion of eDNA in mucus (see Section 4 above). However, its combination with antibiotics has shown enhanced bacterial clearance, as the less viscous mucus exposes previously protected bacteria to antibiotic action. eDNA is a major component of the backbone of the *P. aeruginosa* biofilm matrix and is present in biofilms of other species such as *B. cepacia* complex, *S. aureus*, *A. xylosoxidans*, and *S. maltophilia*, though to a lesser extent.

Studies show that DNase-I combines well with commercially available tobramycin solutions (Tobi^®^ and Bramitob^®^), for inhalation using sulphate as the excipient, but not with Gernebcin^®^-80 mg which was incompatible due to its having 0.05% sodium metabisulphite as the excipient, most likely due to the change in pH in metabisulphite as it degrades to sulphurous acid a process likely to affect DNase-I activity [109]. The DNase-1+Tobi or Bramitob admixtures have proved successful in significantly reducing and sometimes eliminating *P. aeruginosa* by DNA degradation and improved nanoparticle penetration of CF sputum [110]. 

## 6. Emerging Treatments for Bacterial Infection in CF

Is there a need for novel treatments?

The inability of antibiotics to eradicate chronic infection alone has spurred research into novel treatments that target significant features of bacterial physiology such as quorum sensing, biofilm formation and host inflammation. 

### 6.1. Non-Steroidal Anti-Inflammatory Compounds

Inflammation is a long-term event in infection of CF lung, with cytokines, proteases, oxygen radicals and elastase being released continuously once infection is established [111]. This inflammation fails to clear the infection but causes changes to the lung histopathology through a self-perpetuating cycle of airway obstruction, chronic endobronchial infection, and excessive airway inflammation. In vitro studies have indicated a potential role for non-steroidal anti-inflammatory compounds (NSAIDs) as an anti-inflammatory in CF lung and as potential biofilm disruptors. A four-year double-blind, placebo-controlled clinical trial in US CF patients in the 1990s showed that twice-daily high doses of the NSAID ibuprofen, which has specific activity against neutrophils, resulted in a slower rate of decline in % predicted FEV_1_, better preservation of body weight, fewer hospital admissions, and better chest radiograph scores, with no significant adverse effects [112]. At the microbiological level, a recent ibuprofen study showed that 50, 75 and 100 µg/mL ibuprofen significantly reduced (0.5 to 3 log_10_) in a dose-dependent manner the bacterial population for CF isolates of *P. aeruginosa, B. cenocepacia* and *B. multivorans* at 12 h, while repeated treatment at 18 h sustained this reduction [113]. A recent Cochrane review of four published ibuprofen clinical trials concluded that it probably slows the progression of lung disease in children with CF. However, due to likely side-effects associated with long-term administration, it is not recommended in people under 18, and in patients receiving aminoglycoside treatment, to avoid kidney damage [114].

### 6.2. Quorum-Sensing Inhibitors

The central role of QS systems in *P. aeruginosa* and *S. aureus* virulence are well described in the literature, and QS systems have been identified in other bacterial species, including the pathogens *E. coli* O157:H7, *Helicobacter pylori*, *Vibrio cholerae* and *Pseudomonas syringae* [115]. Attempts to identify QS inhibitors through screening of natural products, screening of small molecule libraries, in silico screening, and synthesis of focused libraries based on native autoinducer structures have yielded several candidates for study. Some of these QS inhibitors have been demonstrated to prevent bacterial aggregation and biofilm formation in vitro, but it should be noted that little progress has yet been made with animal or human in vivo studies.

The thiol-containing garlic extract ajoene inhibits the small regulatory RNAs RsmY and RsmZ. Studies showed addition of 100 g/mL ajoene to a *P. aeruginosa* biofilm followed by addition of 10 g/mL tobramycin reduced bacterial viability by >90%, whereas addition of tobramycin or ajoene alone had no effect. Microarray analysis indicated that ajoene’s action was targeted; it was able to achieve its effect by inhibiting a small selection of QS genes rather that the entire regulon [116]. Ajoene decreased expression of both small RNAs in a dose-dependent manner and depleted expression of RsmA, the global regulator (amongst others) of *lasI* and *rhlI*, and the hydrogen cyanide and rhamnolipid biosynthesis genes [117]. Ajoene also showed efficacy against *S. aureus* RNAIII, the effector of QS-induced gene expression encoded by the *agr* QS system, the main QS system of *S. aureus*. Studies using *rnaIII*::*lacZ* reporter fusions demonstrated a dose-dependent relationship with a maximum 77-fold decrease in RNAIII after 240 min of treatment [117]. 

Halogenated furanones, naturally occurring structurally related molecules containing a five-membered heterocyclic furan ring, can selectively attenuate *P. aeruginosa* virulence factors such as *lasB* [118]. Treatment of *P. aeruginosa* PAO1 containing green fluorescent protein reporters of *lasI* and *rhlI* with 200 μL of each of two brominated furanones resulted in 41% and 43% reduction in *lasB* expression [119]. Furanones have also been found to have a sensitizing effect on *P. aeruginosa* persister cells in a way that may influence their antibiotic eradication.

Flavenoids are naturally occurring metabolites containing two phenyl rings (A and B) and a heterocyclic ring. Examples include naringenin, baicalein and quercetin. The presence of two hydroxyl groups in the A-ring is responsible for inhibiting LasR binding to *P. aeruginosa* homoserine lactones. LasR is inhibited through allosteric attachment to the *lasR* ligand binding domain on the homoserine lactone, while some flavonoids, including 7,8-dihydroxyflavone, also partly inhibit RhlR through inhibition of *rhlA* transcription [120]. The result is a reduction in pyocyanin production and swarming in a LasR/RhlR-dependent manner. Quercetin has also been shown to inhibit biofilm formation in *S. aureus* [121,122] with 1 μg/mL decreasing biofilm production by  >50% in two MSSA and one MRSA strain while simultaneously suppressing expression of adhesion related, quorum-sensing, and virulence genes.

A number of other QS inhibitors and their synthetic analogues and derivatives have been investigated in vitro. These include iberin from horseradish, where studies showed 32 and 64 μg/mL iberin significantly downregulated *P. aeruginosa lasB* (−46.8 and −89.8 fold *p* < 0.01) and *rhlA* (−22.2 and −59 fold *p* < 0.01) [123]. Hamamelitannin, an ester of D-hamamelose (2-hydroxy-methyl-D-ribose) derived from the witch-hazel tree, interferes with RNAIII expression in *S. aureus* by blocking/targeting the RNAIII activating protein (TRAP) while concurrently increasing susceptibility of MRSA isolates and their biofilm to vancomycin [124]. 

All Burkholderia species encode at least one QS system, designated CepI, consisting of a homoserine lactone synthase and a homoserine lactone receptor. CepI synthesises two homoserine lactones: N-octanoyl-homoserine lactone (C8-HSL) and, in smaller amounts, of N-hexanoyl-homoserine lactone (C6-HSL). Strains of the ET12 lineage, have four QS systems: CepIR, cciIR, a diffusible signal factor (BDSF)-based system RpfF_BC_ and a non-ribosomal peptide synthetase-like cluster *ham* [125,126]. CepI is involved in biofilm formation, protease production and virulence. 

Certain cyclic dipeptides (2,5 diketopiperazines) have been demonstrated to significantly inhibit CepI. In vitro studies showed four diketopiperazines significantly inhibited protease, siderophore and biofilm production while in vivo experiments with two of these in the *C. elegans* model showed significantly better survival for diketopiperazine-treated worms (*p* < 0.05) [127,128]. Concentrations of 1, 5, 10 and 25 μM diketopiperazine were added to wells of 96-well plates containing *C elegans* with *B. cenocepacia* (infected) and compared to untreated and uninfected controls. At 48 h, 35 ± 18% of untreated controls survived compared to 82 ± 5% and 75 ± 3% at 25 μM of each diketopiperazine tested. Such in vivo testing of QS inhibitors represents a small but significant step to quantifying their potential use. Progression to higher order (mammalian) models is now needed to test clinical efficacy.

### 6.3. Antioxidants and Biofilm Disruption: Glutathione, N-acetylcysteine and Ascorbic Acid

The CF lung suffers from a systemic redox imbalance caused by the inability of cells bearing mutations in their CFTR to efflux glutathione, the most abundant cellular antioxidant, into the extracellular environment. Additional oxidants are released by inflammatory neutrophils at lung infection sites, contributing further to the imbalance. In *P. aeruginosa* biofilms, the metabolite pyocyanin intercalates with and strengthens the eDNA crosslinks within the matrix. Glutathione has been shown to disrupt pyocyanin-mediated virulence by interrupting ROS production [129]. Glutathione is synthesised and maintained at 300–800 µM in the airway surface liquid of healthy lung, but is depleted to less than 10% of that concentration in the airway surface liquid of CF patients, possibly as a result of the combined effect of infection and ROS reactions [130]. 

In the light of these findings, studies have sought to evaluate whether exogenously added glutathione can overcome the effect of pyocyanin and ROS and restore depleted intracellular levels of glutathione. Klare et al. (2016) established the efficacy of glutathione in disrupting the biofilms of both CF and non-CF strains of *P. aeruginosa* [131], while a follow-up study demonstrated the ability of glutathione to restore the cellular health of infected A549 lung epithelial cells [132]. A more wide-ranging study recently investigated its effectiveness on clinical isolates from a range of bacterial pathogens, and found good correlation for the effect of glutathione on CF isolates from previous studies, with the exception of *Klebsiella pneumoniae* biofilms, which displayed a distinct resistance to glutathione disruption [133].

Glutathione has one major drawback in its potential use as an exogenously added antioxidant. It is inherently unstable at temperatures above freezing and is rapidly converted to the oxidised form, glutathione disulphide. Research has thus focused on N-acetylcysteine, a metabolic precursor of glutathione and a more stable redox molecule, to restore reducing capacity to lung epithelia and also disrupt bacterial biofilms.

Treatment with N-acetylcysteine increases intracellular glutathione, thus protecting cells against the neutrophil-driven generation of ROS that mediate chronic tissue damage and fibrosis in CF [134]. Since the late 1960s nebulised N-acetylcysteine has been commonly prescribed to patients with CF, particularly in Europe, to reduce sputum viscosity and improve expectoration. More recently, attention has turned to taking advantage of its effectiveness as an antioxidant. N-acetylcysteine in doses greater than 1800 mg/day were shown to reduce the migration of neutrophils to the lungs and restrict neutrophil access through narrowing of lung capillaries, while simultaneously increasing intra-neutrophil glutathione [135]. A double-blind, placebo controlled clinical trial of 21 patients on 700 mg/day (n 10) and 2800 mg/day (n = 11) for 12 weeks showed that 2800 mg/day was well tolerated [136]. This study measured extracellular glutathione and cytokines in sputum supernatants, as well as total leukocyte numbers and cell differentials in the sputum’s solid phase and found that sputum concentrations of tumour necrosis factor-α (TNF-α) and interleukin-8 (IL8) were not decreased by 2800 mg/mL N-acetylcysteine.

At its intrinsic acidic pH of 2.2, N-acetylcysteine can cause bronchospasm in some patients [137]. However, this can be managed by adjusting the pH to 6–7. The commercially available mucolytic NAC Omegapharm^®^ (Symbion Pharmaceuticals) has a pH of 6–7.5 and is nebulised at a concentration of 200 mg/mL [138].

High doses of N-acetylcysteine were reported to cause pulmonary arterial hypertension (PH) in mice, which mimicked the effects of chronic oxygen deprivation [139]. To determine if N-acetylcysteine treatment was associated with a change in human neutrophil elastase activity while also monitoring for potential PH, Conrad et al. (2015) undertook a 24 week multicentre, randomized, double-blind, proof-of-concept study of 70 CF patients, treated orally with 900 mg N-acetylcysteine thrice daily. A cohort comprising 16 of the patients was separately followed for possible PH (N-acetylcysteine = 8, Placebo = 8) [140]. The results showed that the N-acetylcysteine-treated group maintained baseline % predicted FEV_1_ throughout the 24 week period while a 4–6% decline in FEV_1_ occurred in the placebo cohort. The cohort of 16 patients in the PH group showed no evidence for the development of PH.

While the mucolytic and anti-inflammatory effects of N-acetylcysteine were being quantified in vivo, in vitro research focused on the effectiveness of N-acetylcysteine in disrupting the matrix of bacterial biofilms and killing the exposed cells, usually in combination with an antibiotic. The activity of N-acetylcysteine in promoting dispersal of preformed biofilms could be related either to perturbation of microbial physiology or to a direct effect of N-acetylcysteine in altering the architecture of the matrix [141]. In an early study of the effects of 0.003 to 8 mg/mL N-acetylcysteine alone on 15 *Staphylococcus epidermidis* biofilms, Perez-Giraldo et al. (1997) observed a significant dose-related decrease in viability, with 8 mg/mL resulting in a 74% decrease in viability compared to the untreated control [142]. In a study of the effectiveness of N-acetylcysteine on biofilms of clinical respiratory strains and the lab strain PAO1 of *P. aeruginosa* by Zhao et al. (2010), 6-day-old biofilms were treated with 0, 0.5, 1, 2.5, 5 and 10 mg/mL N-acetylcysteine plus minimum inhibitory concentrations (MIC) of ciprofloxacin for 24 h at 37 °C, and results showed that all N-acetylcysteine-ciprofloxacin combinations significantly decreased viable biofilm-associated bacterial counts relative to the control. Synergy was best achieved at 0.5 mg/mL N-acetylcysteine and 0.5 *×* MIC ciprofloxacin (*p* < 0.01) [143]. Using a GFP-expressing *P. aeruginosa* PAO1 for imaging by confocal laser scanning microscopy the authors showed that fluorescence was almost non-existent after treatment with 10 mg/mL N-acetylcysteine alone, indicating that the antioxidant alone was capable of both disrupting and killing the bacteria at high doses.

**Figure 3:**
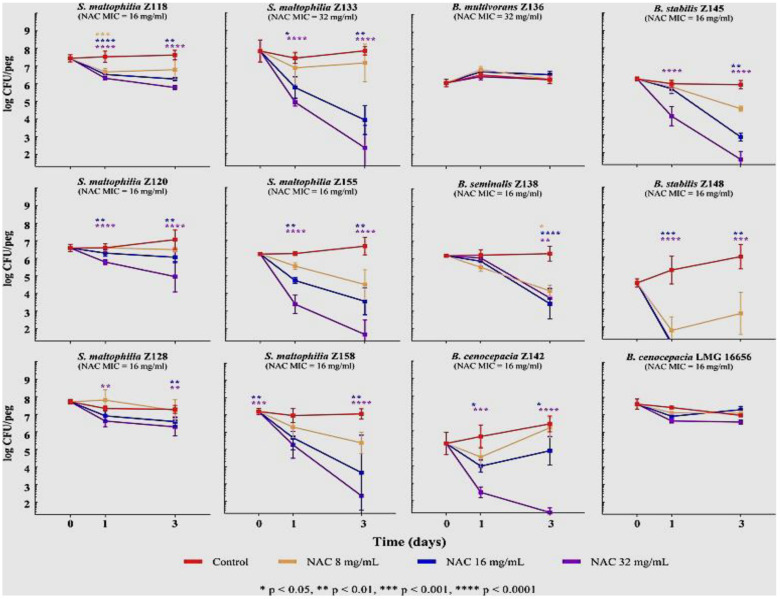
Time-kill curves of N-acetylcysteine for 2-day-old biofilms of *S*. *maltophilia* and BCC. Biofilms were grown on peg lids using the model described by Harrison, J.J. et al. [146]. Data from at least two independent experiments, with at least four replicates per condition per experiment. Median values with standard deviation are plotted. The *x* axis is set at the limit of detection (i.e., 1.3 log CFU/peg). From Pollini, S. et al., 2018, *PLoS ONE* 13: e0203941 [144].

Recent studies have also investigated the efficacy of N-acetylcysteine on other CF bacteria, including *S. maltophilia, B. cepacia* and *S. aureus*. Biofilm and planktonic cultures of 19 *S. maltophilia* and 19 BCC isolates from CF and other sources were treated with N-acetylcysteine in a wide-ranging study by Pollini et al. [144]. Biofilms were grown in the presence of 0, 4, 8 or 16 mg/mL N-acetylcysteine for 72h in daily refreshed CAMB medium, and N-acetylcysteine MICs determined for both species. Time-kill curves for 12 two-day old *S. maltophilia* and BCC (n 4 per treatment), demonstrated a range of survival patterns, with three of six *S. maltophilia* and four of six BCC isolates undergoing significant 3 to 6 log_10_ reductions at 16 and 32 mg/mL N-acetylcysteine while the remainder showed smaller or negligible reductions (Figure 3).

Similarly, testing of N-acetylcysteine against *P. aeruginosa*, *H. pylori*, *S. aureus* and *Streptococcus mutans* showed varying efficacy in biofilm disruption of each species, with N-acetylcysteine being most effective against *P. aeruginosa* biofilms compared to the other species [145]. Biofilms of *P. aeruginosa*, *S. aureus*, and *S. mutans* were grown to 24 h, *E. coli* to 48 h and *H. pylori* to 72 h in 48-well plates and then treated with 80, 40, 20, 10, 5, 2.5, and 1.25 mg/mL N-acetylcysteine. With the exception of *P. aeruginosa*, N-acetylcysteine actually promoted biofilm formation at concentrations of 2.5–10 mg/mL in all other species, indicating a threshold concentration is required for N-acetylcysteine to be effective in these species.

Dry powder formulations of N-acetylcysteine with and without added antibiotic, have also been trialled on biofilms of *P. aeruginosa,* to identify synergistic or additive effects [147]. N-acetylcysteine and the antibiotics were separately dissolved, and mixing was carried out slowly until the correct ratios were achieved. Three separate dry powder formulations were made: azithromycin/N-acetylcysteine, tobramycin/N-acetylcysteine and ciprofloxacin/N-acetylcysteine and tested against 24-h old *P. aeruginosa* biofilms in 96-well plates. Biofilm mass was measured using the crystal violet assay. The results showed that at 10 μg/mL, azithromycin/N-acetylcysteine formulations reduced *P. aeruginosa* biofilm by 40% compared to control, while azithromycin alone reduced biofilm by 55% compared to control. At 0.3 μg/mL, ciprofloxacin/N-acetylcysteine and ciprofloxacin alone both reduced biofilm viability by 88%. Tobramycin/N-acetylcysteine (10 μg/mL) and tobramycin alone both reduced the biofilm viability by 95%. These findings indicated that combined formulations of N-acetylcysteine with azithromycin or ciprofloxacin maintained or enhanced the effect of the antibiotic alone, however tobramycin/N-acetylcysteine formulations were not more effective than tobramycin alone. It should be noted that the concentrations of N-acetylcysteine used in this study were lower than those shown to inhibit biofilm formation in *P. aeruginosa* in vitro in other studies, which have used from 0.5 mg/mL N-acetylcysteine [143] to as much as 80 mg/mL [148] to disrupt biofilms and reduce bacterial viability.

**Figure 4:**
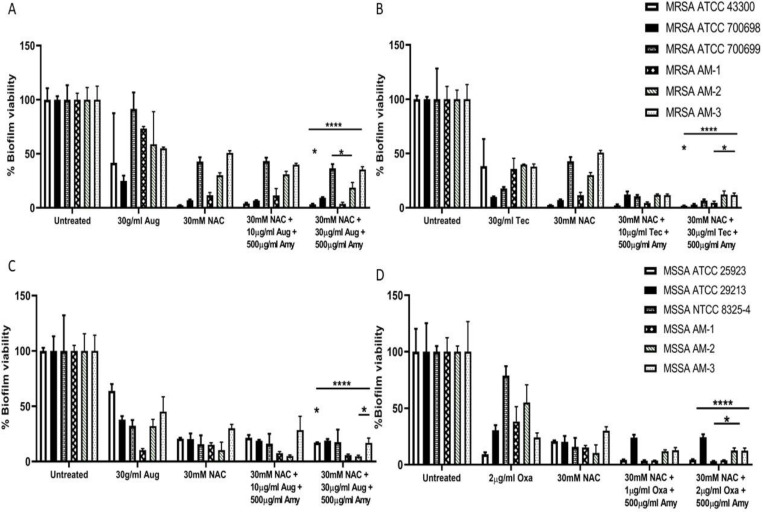
Effect of N-acetylcysteine +/− antibiotic on *S. aureus* biofilm growth. Quantification of viable *S. aureus* biofilm (**A**,**B** = MRSA; **C**,**D**+ = MSSA) after different combination treatments showed that N-acetylcysteine plus antibiotic significantly disrupted MRSA and MSSA biofilms compared to antibiotic alone (*p* < 0.05) and compared to untreated controls (*p* < 0.0001). Data represent mean ± SD of five biological replicates. Tukey’s multiple comparisons test was used to evaluate statistical significance (**** *p* < 0.0001, * *p* < 0.05). Aug, amoxicillin/clavulanate; Amy, amylase; TEC, Teicoplanin; OXA, oxacillin. From Manoharan et al. *J. Antimicrob. Chemother* 2020, 75:1787–1798 [149].

Recent in vitro studies of N-acetylcysteine efficacy on *S. aureus* biofilms of six MRSA and six MSSA isolates demonstrated that N-acetylcysteine is equally disruptive against Gram-positive organisms [149]. Addition of 30 mM N-acetylcysteine alone disrupted the biofilms of all 12 isolates by ≥90%, with a 2–3 log_10_ decrease in CFU/mL. Isolates were sourced from various body sites, including CF sputum, pus, wound and conjunctiva, illustrating that isolation site does not affect the ability of N-acetylcysteine to disrupt *S. aureus* biofilms. Addition of antibiotic (30 μg/mL Teicoplanin or Augmentin for MRSA; 2 μg/mL Oxacillin for MSSA) disrupted the biofilms further (>96%), thus establishing the validity of a combined treatment (Figure 4).

**Figure 5:**
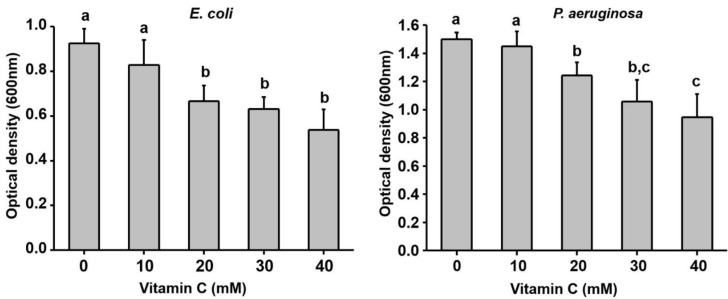
Effect of ascorbic acid on *E. coli* and *P. aeruginosa* biofilm formation. Biofilms were stained with 1% *w*/*v* CV and the OD_600_ measured. All data represent mean ± standard deviation. Columns topped with a different letter are significantly different from each other (*p* > 0.05). From Pandit et al. *Frontiers in Microbiology* 2017, 8:2599 [152].

Ascorbic acid (vitamin C) is another abundant antioxidant that could potentially act as a disruptor in conjunction with an antibiotic, or other compounds such as surfactants. Studies to date have, however, provided conflicting or insufficient evidence of its effectiveness across species and growth conditions. One study on planktonically grown *S. aureus* demonstrated that 5 mM ascorbic acid alone inhibited growth 100% after 12 h incubation [150], while another study of ascorbic acid alone and in combination with rhamnolipid showed no significant disruption of *P. aeruginosa* biofilms [151]. Nevertheless, evidence exists that ascorbic acid inhibits quorum sensing, thus affecting chemotactic behaviour and ultimately biofilm formation. Pandit et al. (2017) subjected 24 h biofilms of *P. aeruginosa, Bacillus subtilis* and *E. coli* to 10 to 40 mM sodium ascorbate at neutral pH. For *P. aeruginosa* and *E. coli*, the OD_600_ of CV-stained biofilms was quantified, while for *B. subtilis*, biofilm biomass and viability, as well as protein, eDNA and EPS matrix, were quantified [152]. The results demonstrated that while 10–40 mM ascorbic acid did not significantly inhibit the planktonic growth of *B. subtilis*, *E. coli*, or *P. aeruginosa*, biofilm formation was significantly impaired for all three species. For *P. aeruginosa* (Figure 5), biofilms decreased by 25% at 20 mM ascorbic acid and 40% with 40 mM ascorbic acid (*p* < 0.05). 

With respect to matrix polysaccharides, proteins and eDNA, *B. subtilis* biofilms showed a reduction of all three components when treated with ascorbic acid. Fluorescent probes were used to visualise EPS and eDNA, and a close correlation was identified between increased ascorbic acid concentration and reductions in both EPS and eDNA, leading to a ‘thinning’ of the matrix.

Successful disruption of *Mycobacterium tuberculosis* biofilms with ascorbic acid has also been reported [153]. *M. tuberculosis* was grown to pellicle stage in 96-well plates over eight weeks and treated with 2.8 mg/mL ascorbic acid (4 *×* MIC), followed by incubations for seven days at 37 °C. This concentration was sufficient to cause the death of the *M. tuberculosis* in the pellicle within the 7 day period. While *M. tuberculosis* is not a common infector of CF lung, the related non-tubercular *M. abscessus* is a low prevalence infector of CF patients [154].

Another likely effect of ascorbic acid is that it alters the regulatory pathways associated with bacterial stress response and dormancy through the stress response regulator guanosine 5′ diphosphate 3′diphosphate ((p)ppGpp). In a study of ascorbic acid on *Mycobacterium smegmatis* biofilms, Syal et al. (2017) found that 50 mM ascorbic acid reduced (p)ppGpp synthesis by >95%, while 100 mM stopped synthesis completely [155]. 

The general consensus emerging from these in vitro antioxidant studies of biofilm disruption and bacterial eradication is positive—they are effective and non-toxic to human cells. The challenge is to now reproduce these findings in in vivo studies.

### 6.4. Silver Nanoparticles

Silver nanoparticles (AgNP) have long been known for their antibacterial properties and have been used as a lining in bandages initially as silver nitrate and subsequently as silver sulphadiazine to prevent infection in recovering burns patients [156,157]. The mechanism of action of silver has not been definitively described, but the evidence indicates it causes bacterial cell membrane damage through production of free radicals leading to perforation and leakage of contents. Silver nanoparticle/silver cations have been shown to bind to the thiol groups of amino acids such as tyrosine. Modulation of the tyrosine within proteins involved in cell cycle progression and in the synthesis of capsular polysaccharides leads to inhibition of protein synthesis and ultimately, cell death. 

One area where AgNP have shown good promise is as an inhibitor of biofilm formation. Radzig et al. (2013) reported significant inhibitory effects by AgNP (av. size 8.3±1.9 nm) on the growth and biofilm formation of three Gram-negative species: *E. coli*, *P. aeruginosa* and *Serratia proteamaculans* [158]. Overnight cultures supplemented with different AgNP concentrations were grown for 24 h in 96-well plates with low shaking. After washing off unattached cells, biofilms were stained and assayed using 1% *w*/*v* crystal violet. The results showed significant biofilm and planktonic growth inhibition in all three species; with 4 ng/μL AgNP resulting in a drop in OD_600_ from ca. 0.3 to 0.005 for *E. coli* and ca. 0.6 to 0.35 for *P. aeruginosa* while 8 ng/μL resulted in almost complete inhibition of both species (OD_600_ ≤ 0.1). *S.* *proteamaculans* was more resistant to AgNP, requiring ca. 20 ng/μL AgNP to reach an OD_600_ ≤ 0.1.

Mohanty et al. (2015) used starch-stabilised AgNP on *S. aureus*, *P. aeruginosa*, *Shigella flexneri* and *Salmonella enterica* (Serovar Typhi), grown as 48 h biofilms in 96-well plates [159]. Different concentrations of AgNP were added to biofilm containing-wells in the plates; the results showed significant killing of each species in a dose-dependent manner, with one hour of incubation resulting in >75% and 92% loss of cell viability at 1 μM and 2 μM AgNP, respectively, while four hours of incubation resulted in >98% loss of cell viability at 2 μM AgNP (*p* ≤ 0.05).

Combinations of AgNP plus antibiotics have been trialled on biofilms. Habash et al. (2014) used AgNP with aztreonam, an antibiotic used to treat *P. aeruginosa* CF infections and disrupt and eradicate *P. aeruginosa* PAO1 biofilms [160]. *P. aeruginosa* biofilms grown to 20 h in 96-well plates were rinsed to remove planktonic cells and re-incubated for a further 20 h with AgNP, AgNP + aztreonam or aztreonam alone at a range of AgNP diameters and aztreonam concentrations. After the second incubation, planktonic cells were removed and OD_600_ measured to determine if a sufficient number of cells were present to re-establish biofilm. Biofilm viability was measured and expressed as a percentage of the no treatment controls. Plates were then re-incubated 20 h in a recovery phase with fresh media to determine biofilm biomass by CV assay. The results showed that treatment with aztreonam alone actually enhanced biofilm recovery post-treatment, and that “size does matter” for AgNP effectiveness. The smaller (10 and 20 nm) AgNP significantly reduced biofilm biomass at concentrations of ≥0.625 µg/mL, while the larger 40 and 60 nm AgNP reduced biofilm biomass at concentrations of ≥2.50 µg/mL. Significant antimicrobial synergistic effects (Bliss coefficient) were also observed with 10 nm AgNP + Azt and 100 nm AgNP +Azt over AgNP alone, with the former being most effective.

In a follow-up study using AgNP with and without tobramycin, Habash et al. were again able to show a significant synergistic effect with smaller AgNP (10 and 20 nm) and tobramycin, this time on CF clinical strains of *P. aeruginosa.* One contrast with the previous study was that tobramycin alone did not lead to biofilm recovery post-treatment, indicating that tobramycin has better inhibitory effect on biofilms in vitro than aztreonam [161].

It is important to note that no mammalian in vivo toxicity studies have been conducted to date on the use of AgNP against respiratory infections. In particular, inhalation of AgNP poses unknown risks. Current research has focused on in vitro assays, and if future mammalian in vivo assays indicate low risks, it may be possible to translate this research into usable medical combinations of AgNP with antibiotics. Cell line studies provide an initial assessment of AgNP toxicity, and one study exposed well-differentiated non-CF and CF human bronchial epithelial cell lines with an established air–liquid interface, and the human bronchial epithelial cell line BEAS-2B, to 20 nm AgNP in the form of nanoaerosols [162]. Perhaps unsurprisingly, addition of AgNP resulted in significantly higher necrosis in a set of markers in the CF cells compared to the normal and BEAS-2B cells.

**Figure 6:**
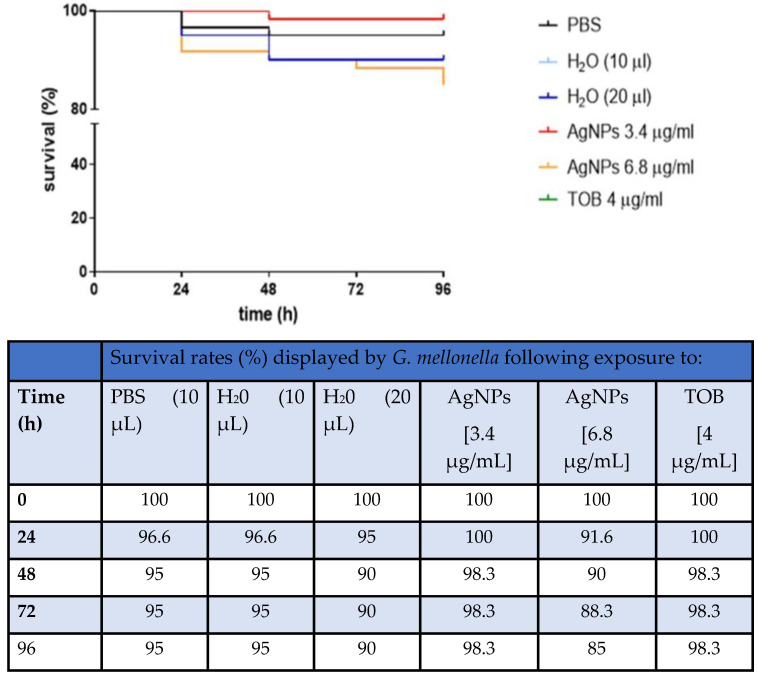
Survival of *Galleria mellonella* larvae after inoculation with AgNP or tobramycin. Larvae (n = 20) were inoculated with 10 or 20 μL of specific AgNP (6.8 and 3.4 μg/mL), or tobramycin (4 μg/mL) and incubated for 96 h at 37 °C. Control larvae were injected with H_2_0 (vehicle), or PBS (to show injection-associated trauma). Larvae were monitored every 24 h for survival until 96 h. Table of survival rates correlates with data points in graph. Results are the average of three biological replicates. From Pompilio et al. *Frontiers in Microbiology* 2018, 9:1349 [163].

Recently, Pompilio et al. (2018) utilised a non-mammalian model, larvae of the wax moth *Galleria mellionella,* to assay AgNP toxicity, as part of larger in vitro study of AgNP disruption of CF pathogen biofilms [163]. Twenty larvae weighing 250–350 mg, were injected with 10 μL of one of: 3.4 μg/mL AgNP, 6.8 μg/mL AgNP; or 4 μg/mL tobramycin and scored every 24 h for 96 h. Control larvae were either inoculated with distilled water only, to simulate nanoparticle trauma, or not inoculated. The results (Figure 6) were encouraging, with survival at the longest exposure time (96 h) being 98.3% for 3.4 μg/mL AgNP and 85% for 6.8 μg/mL AgNP. One significant finding of the in vitro section of this study was that the authors were able to correlate their AgNP findings on three CF pathogens (*P. aeruginosa*, *B. cepacia* and *S. aureus*) with those from another study [164], showing that the two Gram-negatives were more susceptible to AgNP than the Gram-positive *S. aureus*. This suggests that cell wall and biofilm matrix composition may play a role in the effectiveness of disruption and killing by AgNP.

### 6.5. Bacteriophage Therapy

Research into the use of specific bacteriophages (viruses that infect bacteria) as a treatment against infecting bacteria has received new impetus in the last three decades. Bacteriophages (phages) are ubiquitous in water, soil, and animal-associated ecosystems and fall into two categories: lytic phages, capable of entering bacterial cells and causing cell lysis, and lysogenic phages that can integrate their DNA into the bacterial chromosome and express virulence factors [165]. The phages used in therapy fall into the lytic category.

With respect to *P. aeruginosa* biofilms in particular, phages have demonstrated an ability to penetrate into the biofilm and can produce hydrolytic enzymes [166]. This has stimulated interest in their potential use in chronic *P. aeruginosa* infections of CF patients. “Cocktails” of different lytic phages have been developed to counter the fact that CF lungs contain multiple strains and are being increasingly delivered in in vivo studies either as an aerosol or by dry powder inhalation, as opposed to intranasal and intraperitoneal injection. The benefits of aerosol delivery were demonstrated by Semler et al. (2014) who used both aerosol and intraperitoneal injection to deliver a combined therapy of five lytic bacteriophage strains into an immunocompromised mouse model of antibiotic-resistant *Burkholderia cepacia* from CF lung infection, 24 h post-infection. Two days after treatment, a significant decrease (2.5 to 4.0 log; *p* < 0.01) in the mean bacterial load had occurred in aerosol-treated mice but no significant decrease was detected in the load in mice that had undergone intraperitoneal injection [167].

Advances have thus occurred in use of bacteriophages against CF lung infection, with both in vitro and in vivo experiments demonstrating significant reductions in both biofilm and cell viability [168,169,170]. Combinations of bacteriophage and antibiotics such as ciprofloxacin have also shown synergistic effects in in vitro and in vivo work [171,172,173]. There has also been one recent human trial of bacteriophage therapy; Law et al. (2019) administered an FDA-approved emergency use cocktail of four lytic phages to a 26 year-old CF patient who had been taken off the transplant waiting list due to worsening clinical status including acute kidney injury and limited antimicrobial options [174]. Treatment was administered every six hours for eight weeks. At the end of the eight-week course, the patient was on 3 L/min nasal cannula for supplemental oxygen compared to 30 L/min via a heated flow blender at the start of treatment, without sputum production, and was ambulatory. The kidney injury had resolved with a return to baseline renal function.

While significant reductions in load have successfully been achieved with phage therapy, eradication or elimination of infection is still elusive. It also remains to be seen whether some species will develop resistance to the phages being used.

## 7. Attempts to Modify the CF Lung Environment

The following treatments do not have a goal of reducing or eliminating infection, however they have had a side effect of leading to reductions in infection rates and exacerbations in the case of CFTR modifiers or eliminating lung infection for a period of time in the case of lung transplants.

### 7.1. CFTR-Modifying Drugs and Bacterial Infection

The discovery and development of pharmaceuticals that target the genetic cause of CF by attaching and inserting into the CFTR and forcing it into the open functional form, thereby increasing ion transport across the CFTR, are showing great promise in relieving the effects of CF. The end result of this CFTR modification is that the mucus regains its normal ionic composition and is able to be easily removed by coughing. Ultimately, the microbiology of the CF lung is also changed, as the ability of bacteria to infect and maintain colonisation of lung mucus and the underlying epithelium is reduced.

Amongst CFTR mutations, the most common CF-causing allele (present in ~70% of CF patients) is a three base-pair deletion resulting in the loss of a phenylalanine (Phe) residue at position 508 in the amino acid chain (ΔPhe508) and a subsequently misfolded transmembrane regulator. The following mutation sites make up most of the remainder in Caucasian populations: the nonsense mutations Gly542X and Trp1282X (where X is any codon) with premature termination resulting in a truncated protein, the gating mutation Gly551Asp where the CFTR is not activated by AMP or cAMP and is open (gated) for a very short time, and the switch mutation Asp1303Lys [175].

The first CFTR modifier Ivacaftor (Vertex Pharmaceuticals), available since 2012, targets the Gly551Asp gating mutation by increasing the time that the channel is open. Its approval in the US was based on the results of two 48 week Phase 3 clinical trials on 161 CF patients (codenamed STRIVE for children and ENVISION for adults) and a follow-up open-label trial (PERSIST) on the same cohort of either 96 or 144 weeks, depending on the outcome of the former [176]. Measures of key CF baseline indicators including % predicted FEV_1_, weight, and BMI were higher after both STRIVE and ENVISION, with continued gains during PERSIST. The STRIVE and ENVISION placebo groups that went on to receive Ivacaftor in PERSIST also improved performance in these indicators over the course of 96 or 144 weeks of PERSIST. 

Respiratory tract infections and pulmonary exacerbations, however, were two of several adverse effects identified. Respiratory infections in the treatment (non-placebo) cohorts of the STRIVE ENVISION groups rose significantly from 21% of the treatment group at the start of PERSIST to 29% after 144 weeks of treatment (*p* = 0.039—by paired χ^2^ test of proportions in a single sample) while pulmonary exacerbations rose at the same timepoints from 38% to 46% (borderline significant: *p* = 0.052). 

At least one study of CF-specific bacterial species during Ivacaftor use indicates an initial decline in *P. aeruginosa* incidence. In a study of *P. aeruginosa* incidence by culture on 151 CF patients being treated with Ivacaftor by Heltshe et al. (2015), it was found that 29% of those who were *P. aeruginosa*-positive in the year prior to Ivacaftor use were culture negative the year following treatment, while 88% of those that were *P. aeruginosa* free in the year before remained uninfected in the year after [177]. A retrospective cohort study of 276 Ivacaftor recipients compared to 5296 non-recipients showed significant long-term declines in all species except BCC. With regard to *P. aeruginosa*, a reduced incidence with Ivacaftor was identified. However, those who acquired *P. aeruginosa* while receiving Ivacaftor had poorer lung function at baseline [178].

There do not appear to be major changes to the composition of the airway microbiota during Ivacaftor treatment. One study investigated 64 genera for changes in relative abundance before initiation of treatment and sampled every two months after initiation for a total of 13 months [179]. Abundance was quantitated with qPCR, and bacterial community composition was analysed by 16S rRNA sequencing. The 16S data identified 129 operational taxonomic units (OTU) representing the 64 genera, but interestingly *P. aeruginosa* was not identified as a core species (defined as: OTUs present in at least 50% of samples), as only 1 OTU assigned to *P. aeruginosa* was found in five samples obtained from three patients, and only one sample had a relative abundance of ≥1%. As only three relatively young patients were sampled in this study, the numbers and abundance cannot be considered representative of the wider CF population. However, changes were identified in anaerobe populations as a result of Ivacaftor treatment that may have implications for CF lung infection and these changes need to be confirmed in a more comprehensive study.

A recently published study used 16S rRNA sequencing to measure the total and specific bacterial load in the sputum of 31 CF patients aged ≥10 years undergoing Ivacaftor treatment, with an FEV_1_ of at least 40% predicted [180]. In this case, one sample was collected before commencement of treatment and another six months after completion of treatment. While the FEV_1_ improved in the cohort, there was no significant change identified in either bacterial load or bacterial diversity after six months of treatment.

LUM/IVA, a combination of Lumacaftor, a CFTR corrector protein, and Ivacaftor as the potentiator, (marketed as Orkambi) has been available since 2015 to treat patients homozygous for the Δ508Phe mutation and the gating mutation G511Asp. In the US, FDA approval was based on two 24 week trials of 1108 patients (TRAFFIC and TRANSPORT) with Orkambi that resulted in statistically significant (2.6% to 4%) individual improvements in % predicted FEV1, (*p* < 0.001 for all comparisons), as well as a 30–39% reduction in pulmonary exacerbations (pooled rate ratio compared with placebo = 0.61, 95% confidence interval = 0.49–0.76, *p* < 0.001) [181]. Subsequent subgroup analyses demonstrated that the reduced exacerbation rate favoured LUM/IVA therapy over placebo irrespective of patient baseline characteristics including % predicted FEV1, age, sex, medication use, and *Pseudomonas aeruginosa* status [182].

A recent study by Tesell et al. (2019) of exacerbation rates in 21 CF patients with high severity of illness receiving LUM/IVA found no statistically significant difference in the rate of pulmonary exacerbation events (45–48 events, *p* = 0.69), the number of exacerbation days per year (113.31–125.17 exacerbations, *p* = 0.55), and in the annualised exacerbation rate (4.37–4.66 per year *p* = 0.69) in the six months before and six months after treatment started. The overall rate of pulmonary exacerbations was much higher than in the TRAFFIC and TRANSPORT study (4.66 per days per 365 days compared to 0.71 per 336 days) most likely due to severity of illness being greater in the latter [183]. While not pursued further in this study, severe illness indicates lung damage due to chronic bacterial infection, indicating worse outcomes for this subgroup despite LUM/IVA treatment.

With a subset of people still not significantly responsive to dual therapy due to the Δ508Phe mutation introducing multiple defects, a triple combination of modulators comprising two CFTR correctors elexacaftor and tezacaftor, plus ivacaftor as potentiator, underwent phase 3 clinical trials in 2018 in a study funded by the manufacturer (Vertex) to determine if further CFTR correction would lead to significant clinical improvements. Four weeks of treatment of 107 participants, of whom 55 received triple therapy and 52 dual therapy showed significantly improved % predicted FEV1 and sweat chloride concentration in the triple therapy group compared to dual therapy [184]. 

**Figure 7:**
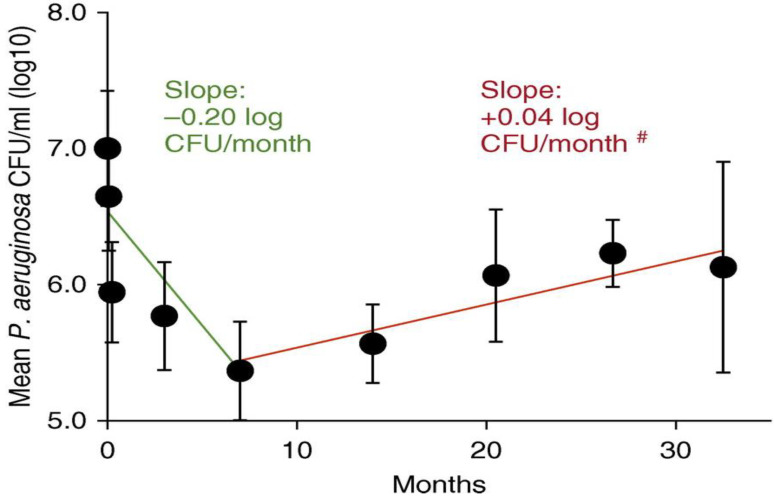
Mean *P. aeruginosa* CFU/mL increase after 210 days of Ivacaftor treatment. CFU values were log transformed and averaged; error bars indicate SEM. Piecewise, mixed-effect linear regression models were used to estimate and test log10 CFU slope changes before and after day 210; # *p* < 0.0001. CFU = colony-forming units. With permission of the American Thoracic Society. Copyright © 2021 American Thoracic Society. All rights reserved. Hisert, K. et al. 2017 *Am J Respir Crit Care Med* 195, 1617–1628 [185]. The American Journal of Respiratory and Critical Care Medicine is an official journal of the American Thoracic Society. Readers are encouraged to read the entire article for the correct context. The authors, editors, and The American Thoracic Society are not responsible for errors or omissions in adaptations.

The lack of consensus with regard to ongoing bacterial infection in the above studies may be due to a number of factors, including cohort size, severity of CF illness, whether live bacteria were counted as opposed to their 16S rRNA and in what stage of disease patients were sampled. With respect to infection, a 2017 study that looked at 12 CF patients over 2 years of Ivacaftor treatment may provide some clarity [185]. All subjects were chronically infected adults; eight were chronically infected with *P. aeruginosa*, two were chronically infected with Burkholderia sp. and two with *S. aureus*, giving this cohort a more typical adult infection profile. A critical finding was that all subjects reduced their sputum *P. aeruginosa* load in the first year, with sputum *P. aeruginosa* CFUs beginning to decrease at day 2 of treatment. Average sputum *P. aeruginosa* CFUs had declined significantly by 10-fold (*p* = 0.012) after one week. However, CFUs levels rebounded after 210 days and remained high in six of seven remaining *P. aeruginosa* infected patients (Figure 7). Most tellingly, none of the subjects eradicated their *P. aeruginosa* during the two-year study period, which was the same strain they were infected with prior to commencement of treatment. The overall message from this study appears to be that CFTR modifiers will have specific effects on bacterial loads at specific time points during treatment, however the original strain of bacteria remains in sufficient numbers to recover further down the track.

### 7.2. Lung Transplant and Reinfection

Lung transplantation has been an established therapy for CF patients with end-stage lung damage since the late 1980s. Lung transplants worldwide have been shown to prolong CF patient life expectancy by 10 years in approximately 45% of recipients [186]. Mortality is primarily due to failure of the transplant due to chronic lung allograft dysfunction. However, sepsis and malignancy are also evident in many cases.

There are a number of relative contraindications towards a particular patient having a lung transplant. The microbiological contraindication is chronic infection with virulent or resistant microbes, including *B. cenocepacia*, *Burkholderia gladioli*, and multidrug–resistant *M. abscessus*. These are relative to treatment, therefore if the infection is “sufficiently treated” (i.e., eradicated) preoperatively and there is a reasonable expectation for adequate control postoperatively, the transplant can proceed [187]. Preoperative infection with *B. cenocepacia* has been linked to a poorer prognosis after transplantation compared to CF patients without this infection. One study of postoperative CF patients showed that of 12 patients with preoperative confirmed *B. cenocepacia*, nine died within 12 months of the transplant, eight of these from *B. cenocepacia* sepsis [63]. Poor prognosis as also been documented in cases of preoperative infection with *M. abscessus*, though there are studies demonstrating better than expected outcomes. Lobo et al. (2013) examined the clinical outcomes for 13 CF lung transplant patients with preoperative *M. abscessus*, finding that 50% were still alive after 5 years. The three patients that developed *M. abscessus*-related complications postoperatively were successfully cleared of the infection [188].

Infection with a new Gram-negative bacterial strain, rather than re-colonisation with a previously infecting strain, is also of concern for CF lung transplants as it is believed to pose a greater risk of chronic lung allograft dysfunction; while persistent reinfection with *P. aeruginosa* has been shown to increase the prevalence of the disease Bronchiolitis obliterans [189]. In a study by Orfanos et al. (2018), 15 bacterial species (including *P. aeruginosa*, *S. aureus*, *H. influenzae*, *A. xylosoxidans*, *M. abscessus,* and *S. maltophilia*) were tracked by examining sputum before and after transplant, while a sample from a subgroup of patients was further characterised via MALDI-TOF [190]. Forty patients provided both preoperative and postoperative sputum samples (bacterial sputum culture, bronchial aspirates or broncho-alveolar lavage), with at least two samples taken at 1, 6 or 12 months after the transplant. Preoperative infection with *P. aeruginosa* was detected in 36 (90%) of the patients, while the remainder had other Gram-negative species. Postoperatively, 17 (51.5%) patients had been re-colonised with the same Gram-negative species at 12 months, 15 of these with *P. aeruginosa*. Eight new strains were also identified. Interestingly, six of these were Gram positive *S. aureus* strains and only one was *P. aeruginosa*, indicating low levels of new strain infection but relatively high levels of same-strain re-colonisation by Gram-negatives, mainly *P. aeruginosa*, a result that augurs well for the likelihood of transplant patients avoiding chronic lung allograft dysfunction.

The conclusion gleaned from these studies is that while lung transplants are a regular feature of treatment, there are areas for continued concern regarding bacterial infection. These fall into two categories: Transplant contraindications for prior infection with *B. cenocepacia* in particular, and postoperative infections with Gram-negative species leading to increased mortality from chronic lung allograft dysfunction and Bronchiolitis obliterans.

## 8. Summary—Treatment of Bacterial CF Infections 

The control and eradication of bacterial infection in the lungs of people with CF has benefited from advanced antibiotic-use regimens coupled with mucolytics such as DNase-I, regular physical therapy, lung transplantation and more recently through CFTR-modifying drugs. The infecting species have different pathogenic mechanisms at their disposal and differing prevalence levels within age groups, nevertheless progress is being made against all significant infectors. Problems of persistence and reinfection continue to exist, particularly with prolific biofilm forming species such as *P. aeruginosa*. Novel emerging treatments to break down biofilms and reduce or eradicate bacterial infection have introduced new compounds such as antioxidants that act to disrupt the biofilm matrix, with often excellent in vitro and in vivo (animal) model outcomes. Much remains to be done to translate and adapt these novel treatment findings to human clinical use, though there is evidence of progress on this front, with N-acetylcysteine trials on CF patients showing good promise [144,149].

Successful in vivo trials of an antibiofilm therapy offer a way forward for a comprehensive approach to eradicating CF infection, where a CFTR modifier drug could be coupled with the most successful of the novel antibiofilm treatments. The evidence presented in Section 7 showed a definite need for additional infection prevention measures that can complement treatment with CFTR-modifiers over the longer term. Regular use of an antibiofilm combination therapy containing N-acetylcysteine and an appropriate antibiotic where the components have been demonstrated to work synergistically, plus DNase-I against *P. aeruginosa* biofilms, may enable people on CFTR-modifiers to remain largely infection free for the majority of their life.

There are CFTR mutations that cannot currently be modified by the currently available drugs, and in these cases, lung transplants will remain the mainstay of long-term treatment in the foreseeable future. However, as infection with *B. cenocepacia* and *B. gladioli* in particular are contraindicated for lung transplants, a preoperative course of antibiofilm combination therapy in these cases can result in bacterial eradication, and a subsequent lung transplant with much better odds for long-term survival. Postoperative infections and biofilms could also be eradicated with the same antibiofilm treatment.

## Figures and Tables

**Table 1:** Change in virulence factor expression over time in infants and young children.

	Part 3: Time since Isolation of Initial Strain (Adapted from Manos et al. 2013 *Eur J Clin MicrobiolInfect Dis* 32:1583–1592 [39].									
**Virulence Factor**	**Units**	**0 Months**	**1–11 Months**	***p* Value ^a^**	**12–23 Months**	***p* Value ^a^**	**24–35 Months**	***p* Value ^a^**	**+36 Months**	***p* Value ^a^**
Pyocyanin	%	18.03	20.00	0.822	19.05	0.918	0.00	0.061	33.33	0.202
Pyoverdine	%	40.98	26.67	0.180	25.40	0.171	18.18	0.166	26.67	0.309
Swarming	%	13.7	11.1	0.725	4.8	0.292	18.20	0.695	8.9	0.552
Elastase	mm^2^	89.88	79.36	0.203	89.20	0.942	100.84	0.367	49.75	0.000 ***
Rhamnolipid	mm^2^	62.07	59.60	0.690	60.23	0.794	66.77	0.607	66.93	0.545
PLC ^b^	mm^2^	97.72	88.68	0.287	95.62	0.827	96.20	0.903	65.56	0.003 **
Haemolysin	mm^2^	96.88	76.96	0.147	83.34	0.385	90.92	0.767	59.88	0.037 *
Total protease	mm^2^	91.50	84.34	0.249	88.99	0.722	94.35	0.755	73.82	0.028 *
Biofilm mass	% ^c^	95.07	70.61	0.033 *	92.71	0.850	100.82	0.721	85.02	0.480
Swimming	mm^2^	78.01	61.71	0.034 *	68.68	0.284	75.34	0.812	58.90	*0.054* †
Twitching	mm^2^	70.96	62.13	0.354	45.34	0.018 *	55.89	0.282	42.67	*0.054* †
Colony size	mm	2.86	2.83	0.874	2.43	0.027 *	2.64	0.375	1.93	0.000 ***

^a^ Wald statistic; *p* values obtained from unadjusted regression analysis; ^b^ phospholipase C; ^c^ % of *P. aeruginosa* PAO1 biofilm grown concurrently. * *p* < 0.05; ** *p* < 0.01; *** ***p* < 0.001**; † borderline *p* < 0.05.

## Data Availability

Not Applicable.
